# The EJC disassembly factor PYM is an intrinsically disordered protein and forms a fuzzy complex with RNA

**DOI:** 10.3389/fmolb.2023.1148653

**Published:** 2023-03-30

**Authors:** Deepshikha Verma, Veena Hegde, John Kirkpatrick, Teresa Carlomagno

**Affiliations:** ^1^ Laboratory of NMR-based Integrative Structural Biology, Centre for Biomolecular Drug Research (BMWZ) and Institute of Organic Chemistry, Leibniz University Hannover, Hanover, Germany; ^2^ Laboratory of Integrative Structural Biology, School of Biosciences, College of LES, University of Birmingham, Birmingham, United Kingdom

**Keywords:** protein-RNA complex, intrinsically-disordered protein, mRNA localization, *oskar* SOLE RNA, PYM

## Abstract

The discovery of several functional interactions where one or even both partners remain disordered has demonstrated that specific interactions do not necessarily require well-defined intermolecular interfaces. Here we describe a fuzzy protein–RNA complex formed by the intrinsically unfolded protein PYM and RNA. PYM is a cytosolic protein, which has been reported to bind the exon junction complex (EJC). In the process of *oskar* mRNA localization in *Drosophila melanogaster*, removal of the first intron and deposition of the EJC are essential, while PYM is required to recycle the EJC components after localization has been accomplished. Here we demonstrate that the first 160 amino acids of PYM (PYM^1–160^) are intrinsically disordered. PYM^1–160^ binds RNA independently of its nucleotide sequence, forming a fuzzy protein–RNA complex that is incompatible with PYM’s function as an EJC recycling factor. We propose that the role of RNA binding consists in down-regulating PYM activity by blocking the EJC interaction surface of PYM until localization has been accomplished. We suggest that the largely unstructured character of PYM may act to enable binding to a variety of diverse interaction partners, such as multiple RNA sequences and the EJC proteins Y14 and Mago.

## 1 Introduction

The localization of mRNA is an evolutionarily conserved process needed for spatial and temporal control of protein expression within the cell. For example, mRNA localization mediates confined protein expression during embryonic development, helping to sustain cell polarization and promote further differentiation. mRNA localization is essential for all developmental processes, such as cell movement, cell specialization and asymmetric cell division (St Johnston, 2005; Besse and Ephrussi, 2009). The absence of localized protein expression can result in aberrant patterning of the embryo and developmental failure.

**FIGURE 1:**
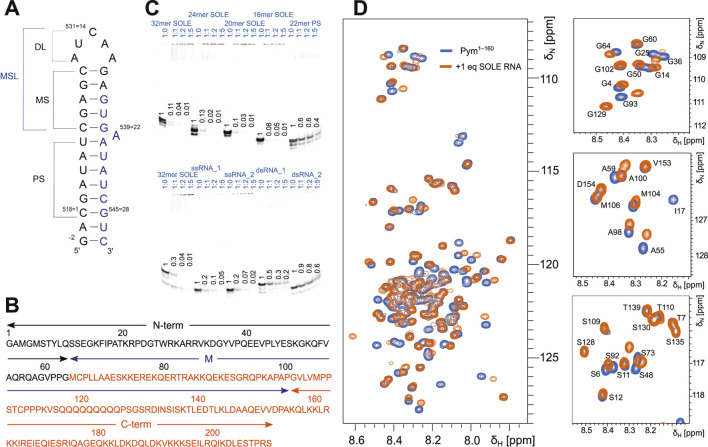
The SOLE RNA binds specifically to PYM^1–160^. **(A)** Schematic of the secondary structure of the SOLE RNA as determined in (Simon et al., 2015). PS = proximal stem; MS = medial stem; DL = distal loop; MSL = medial stem–loop. The nucleotides in black and blue belong to exons I and II, respectively. **(B)** PYM sequence with the definition of the N-terminal (N-term, black), middle (M, purple) and C-terminal (C-term, orange) domains. **(C)** Electrophoretic mobility shift assays (EMSAs) of various RNA constructs in the presence of PYM^1–160^. Each lane contains 65 pmol of RNA dissolved in 10 μL. The protein was added to the RNA in the RNA:protein ratio indicated above each lane. A portion of the RNA is Cy5-labeled at the 5′-end for visualization. The amount of unbound RNA was quantified in each lane and scaled with respect to the amount present in the control lane without PYM. The quantification is given in the figure for each of the lanes. **(D)** Overlay of the ^15^N-HSQC spectra of 120 μM PYM^1–160^ in isolation (blue) and in the presence of an equimolar concentration of 32mer SOLE RNA (orange). Left: complete amide region. Right: expansions of selected regions.

In the oocyte of *Drosophila melanogaster* (*Dm*), *oskar* mRNA localizes to the posterior pole (Brendza et al., 2000; Zimyanin et al., 2008; Martin and Ephrussi, 2009) and has been extensively studied as a model system for mRNA localization. Localization of *oskar* mRNA determines the position of abdomen and primordial germ cells during embryonic development. Splicing of the first two exons and the concomitant deposition of the Exon Junction Complex (EJC) approximately 20–24 nucleotides (nt) upstream of the exon-1–exon-2 junction is essential for *oskar* localization (Ghosh et al., 2012). Upon splicing, a stem-loop RNA element is formed—the SOLE (spliced *oskar* localization element) RNA—which consists of 18 nt from exon-1 and 10 nt from exon-2 (Figure 1). *In vivo* mutational analysis established that the secondary structure, but not the sequence, of the short proximal stem (PS) is important for localization (Ghosh et al., 2012) (Figure 1); mutant SOLE RNAs with alterations in the medial stem-loop (MSL) region supported *oskar* localization with similar efficiency as the wild-type RNA (Figure 1).

The structure of the SOLE RNA has been solved by solution-state NMR spectroscopy in aqueous solution (PDB entry: 5a18) (Simon et al., 2015). This experimental structure corrected the original structural prediction (Ghosh et al., 2012) by showing that the SOLE RNA forms two stems of six (PS, proximal stem) and five (MS, medial stem) base pairs (bp), stacked upon each other (Figure 1A). A22 is bulged out at the junction between the PS and MS (Simon et al., 2015). The presence of non-canonical base pairs in the MS causes a substantial widening of the major groove; in addition, dynamics studies revealed that nucleotides 7–22 of the MS undergo exchange between the major conformation described in (Simon et al., 2015) and a second minor conformation.

The protein known as “Partner of Y14 and Mago” (PYM, Figure 1B) is a cytosolic protein. In humans, PYM is often found in complex with the 40 S ribosomal subunit, where it functions to enhance the translation of spliced mRNA by disassembling the EJC during the first rounds of translation (Diem et al., 2007; Gehring et al., 2009). In agreement, the N-terminal domain of PYM has been reported to bind to the Mago–Y14 heterodimer of the EJC (PDB entry: 1rk8) (Bono et al., 2004). In HeLa cells, PYM overexpression results in an apparent inhibition of EJC association with spliced mRNA, whereas its depletion leads to accumulation and impaired recycling of EJC (Gehring et al., 2009). PYM mutants that are unable to bind the Mago–Y14 dimer are also unable to disassemble the EJC. In *Drosophila*, PYM does not interact with the ribosome and its function as an EJC dissociation factor is independent of translation (Ghosh et al., 2014). Over-expression of *Dm* PYM during oogenesis results in *oskar* mRNA mislocalization. This phenotype was shown to depend on the relative amounts of PYM and *oskar* mRNA; furthermore, mislocalization of *oskar* mRNA upon increased levels of PYM is mediated by the PYM N-terminal and central M domains, while the C-terminal domain acts to partially suppress this mislocalization (Figure 1B) (Ghosh et al., 2014). On the other hand, depletion of PYM does not impair localization of *oskar* mRNA, suggesting that PYM is not required for mRNA transport. The ability of PYM to impact localization has been attributed to its function as EJC disassembly factor, but the precise interplay between PYM, *oskar* mRNA and the EJC has not been well understood.

Here, we determine the structure of PYM^1–160^ in solution and show that the protein is predominantly disordered, but tends to adopt β-sheet-like structures in the first 60 amino acids of the N-terminal domain and forms a more stable helical structure in the region 67–86. Using gel-mobility assays and NMR chemical-shift perturbation (CSP) data, we demonstrate that PYM^1–160^ binds the SOLE RNA, as well as many other RNAs with both double-stranded and single-stranded structures. The interaction appears to be largely independent of the RNA sequence. Critically, the PYM–RNA complex is incompatible with the binding of PYM to the Mago–Y14 dimer, which engages the same PYM surface as RNA. We propose that the PYM–RNA complex serves to sequester PYM from fulfilling its role in EJC disassembly by blocking its canonical interaction with Mago–Y14, thereby preserving the EJC until *oskar* localization is complete. At the same time, the interaction of PYM with the RNA may contribute to ensure that PYM is localized together with the mRNA and the EJC. Our model implies that at end of the localization process PYM is released from the RNA to bind the Mago–Y14 heterodimer. This may happen through a conformational change of the mRNA and/or the EJC triggered by an as yet unknown event. While we started this investigation with the hypothesis that the SOLE RNA element is critical for PYM recruitment, we have found that the SOLE RNA is not a unique RNA binding partner of PYM but other double-stranded RNAs bind PYM with similar affinities. Thus, the role of the specific SOLE RNA element remains to be determined.

## 2 Materials and methods

### 2.1 Protein expression and purification

PYM^1–160^ was cloned into the pET-M11 vector (EMBL plasmid library) with a cleavable His_6_-tag at the N-terminal end. The DNA was transformed into BL21 (DE3) cells and grown in LB media at 37°C. Protein expression was induced by adding 1 mM of IPTG at an O.D. of 0.8; the cell were allowed to grow at 16°C overnight and were harvested by centrifugation at 5,000 g for 20 min at 4°C.

The bacterial cell pellet was resuspended in lysis buffer (50 mM Tris, pH 7.0, 50 mM NaCl, 2.5 mM β-mercaptoethanol, 50 mM glycerol, 10 mM imidazole and protease inhibitor cocktail), sonicated on ice (for 15 min at 50% amplitude with 5 sec-on and 8 sec-off pulses) (Bandelin sonicator with KE76 probe) and centrifuged at 18,500 rpm using an ultracentrifuge (Beckmann Allegra). The supernatant was collected, loaded on a Ni-NTA column (Histrap HP, 5 mL, Cytiva) and washed at least 3 times with 3 column volumes (CVs) of 500 mM LiCl and lysis buffer, alternately, to remove potentially bound nucleic acids. The protein was then eluted with a gradient of 0–500 mM imidazole over 4 CVs. The absence of contaminating nucleic acids was confirmed by evaluating the ratio of the protein sample absorption at 280 and 260 nm. In the next steps, the protein was desalted using a desalting column 26/10 (Cytiva), loaded on an heparin column (Hitrap Heparin HP, 5 mL, Cytiva) and eluted with buffer containing 50 mM Tris, pH 7.0, 2.5 mM β-mercaptoethanol, 50 mM glycerol and 1 M NaCl, followed by desalting and overnight cleavage with TEV protease (1:25 ratio) in the presence of 2 mM dithiothreitol (DTT). The protein was then purified by reverse His-trap, and further by size-exclusion chromatography in buffer containing 50 mM Tris, pH 7.0, 50 mM NaCl, 2.5 mM β-mercaptoethanol, 50 mM glycerol, using an S75 10/300 column (Cytiva). The protein was tested for RNAse contamination before further experiments using the RNAse Alert test kit (Applied Biosystems).

### 2.2 *In-vitro* RNA transcription

SOLE RNA was prepared by *in-vitro* transcription using T7 polymerase produced in-house and a DNA template cloned in pUC19. Before large-scale RNA transcription, the concentrations of DNA, nucleoside triphosphates (NTPs) (Carl Roth), MgCl_2_ and T7 polymerase were optimized to maximize the yield. The RNA was purified by denaturing 12% polyacrylamide gel electrophoresis. To obtain homogeneous 3′ ends, trans-acting hammerhead RNA was used to cleave the SOLE RNA at a well-defined position (Simon et al., 2015). The RNA was resuspended in 50 mM bis(2-hydroxyethyl)amino-tris(hydroxymethyl)methan (Bis-Tris), pH 6.0, 50 mM 2-(N-morpholino)ethane sulfonic acid (MES), 50 mM NaCl, and 2.5 mM *tris*(2-carboxyethyl)phosphine (TCEP).

### 2.3 RNA constructs for EMSA and NMR titrations

The following RNA constructs were designed and obtained from Sigma for NMR titrations and EMSAs (electrophoretic mobility shift assays). The purity of the Cy5-labelled RNAs varied slightly from batch to batch with some RNA preparations showing the presence of shorter oligonucleotides (Figure 1).

SOLE RNA (32mer):

5′-Cy5-GACGAUAUCGAGCAUCAAGAGUGAAUAUCGUC-3′

5′-GAC​GAU​AUC​GAG​CAU​CAA​GAG​UGA​AUA​UCG​UC-3′

26mer SOLE RNA:

5′-Cy5-GAUAUCGAGCAUCAAGAGUGAAUAUC-3′

24mer SOLE RNA:

5′-AUA​UCG​AGC​AUC​AAG​AGU​GAA​UAU-3′

22mer SOLE RNA:

5′-Cy5-GAUCGAGCAUCAAGAGUGAAUC-3′

20mer SOLE RNA:

5′-GAU​CGA​GCA​UCA​AGA​GUG​AAU​C-3′

16mer SOLE RNA:

5′-Cy5-CGAGCAUCAAGAGUGA-3′

5′-CGA​GCA​UCA​AGA​GUG​A-3′

22mer PS RNA (PS stem of SOLE):

5′-Cy5-GACGAUAUCUUCGGAUAUCGUC-3′

SOLE RNA MSL mutant (32mer).

5′-Cy5-GACGAUAUCACACAUCAAACACGAAUAUCGUC-3′

dsRNA_1:

5′-Cy5-GCGACCUGAGG-3′

5′-GCGACCUGAGG-3′

dsRNA_2:

5′-Cy5-GCAUCGAAG-3′

dsRNA_1 (complementary strand):

5′-CCUCAGGUCGC-3′

dsRNA_2 (complementary strand):

5′-CUUCGGGGC-3′

ssRNA_1:

5′-Cy5-GCAUAAGAAAUU-3′

ssRNA_2:

5′-Cy5-GCAUAAGAAAGG-3′

5′- GCAUAAGAAAGG-3′

### 2.4 Electrophoretic mobility shift assays (EMSAs)

For binding assays, we used commercially procured 5′-end Cy5-labelled and unlabelled RNAs. In each reaction, we used a total of 65 pmol of RNA. The RNAs were dissolved in reaction buffer (50 mM Bis-Tris, pH 6.0, 50 mM MES, 50 mM NaCl, and 2.5 mM TCEP). As the efficiency of Cy5-coupling was different for each RNA, we used a different amount of labelled RNA for each RNA construct, to reach similar intensities of the fluorescence signal. We then complemented the amount of labelled RNA with unlabelled RNA to reach a final amount of 65 pmol. The RNAs were annealed by heating at 94°C for 3 min followed by gradual cooling to room temperature. Increasing amounts of PYM^1–160^ were added to reach RNA:PYM^1–160^ concentration ratios of 1:0, 1:1, 1:2 and 1:5 (Figure 1). The final reaction volume was 10 μL. The mixtures were incubated at room temperature for 30 min. 2 μL of 6x-loading buffer (0.2% bromophenol blue and 50% glycerol) were added before loading the reaction mixture on a 12% native gel, which was run at 8 W for ∼2.5 h at 4°C (Hellman and Fried, 2007). The gel fluorescence was imaged at 670 nm using a ChemiDocTM MP Imaging System (Bio-Rad) (GE Healthcare). The images were analysed using the software ImageJ (Schneider et al., 2012).

### 2.5 NMR titrations of PYM^1–160^ with different RNA constructs

The backbone amide resonances of 30 μM of ^15^N-labelled PYM^1–160^ in buffer containing 50 mM Bis-Tris, pH 6.0, 50 mM MES, 50 mM NaCl, and 2.5 mM TCEP were monitored in ^15^N-HSQC spectra in the presence of increasing concentrations of RNA. The chemical-shift perturbations (CSPs) were calculated according to the formula:

$$CSP=\sqrt{{\left({∆\delta }_{H}\right)}^{2}+{\left(0.15∙{∆\delta }_{N}\right)}^{2}}$$

where ${∆\delta }_{H}$ and ${∆\delta }_{N}$ are the chemical-shift differences between the RNA-bound and unbound states in the ^1^H and ^15^N dimensions, respectively.

The spectra were recorded on a 600 MHz Bruker Avance III HD spectrometer equipped with an N_2_-cooled triple-resonance probe-head. The spectra were processed in Topspin 3.2 (Bruker) and analysed in CcpNmr v2.4 (Vranken et al., 2005).

### 2.6 NMR assignments and structure elucidation

Uniformly ^15^N- or ^13^C, ^15^N-labelled PYM^1–160^ was prepared and dissolved in 50 mM Bis-Tris, pH 6.0, 50 mM MES, 50 mM NaCl, and 2.5 mM TCEP and 90%:10% H_2_O:D_2_O. NMR experiments were recorded at 293 K on 600-MHz and 850-MHz Bruker Avance III-HD spectrometers equipped with inverse HCN cryogenic probeheads (nitrogen-cooled and helium-cooled, respectively) and running Topspin 3.2 software (Bruker).

2D ^15^N-HSQC spectra were recorded using States-TPPI for frequency discrimination, with water suppression achieved *via* a combination of WATERGATE and water flip-back pulses to preserve the water magnetization (Bodenhausen and Ruben, 1980; Piotto et al., 1992; Sklenar et al., 1993). 2D ^13^C-HSQC spectra for assignment purposes were recorded with gradient coherence-order-selection and constant-time ^13^C chemical-shift evolution (Santoro and King, 1992; Vuister and Bax, 1992).

Backbone assignments were obtained from 3D HNCO, HN(CA)CO, HNCACB, HN(CO)CACB experiments (Ikura et al., 1990; Kay et al., 1990; Bax and Ikura, 1991; Clubb et al., 1992; Grzesiek and Bax, 1992; Wittekind and Mueller, 1993), recorded using semi-constant-time chemical-shift evolution in the ^15^N dimension to maximize resolution. In addition, HNCACB and HN(CO)CACB experiments were recorded with constant-time chemical-shift evolution in the ^13^C dimension (constant-time period = 28.6 ms). Aliphatic side-chain assignments were obtained from 3D H(CCCO)NH (Logan et al., 1992; Montelione et al., 1992) and H(C)CH-TOCSY (Bax et al., 1990; Kay et al., 1993) experiments. Aromatic side-chain assignments were obtained from 2D (HB)CB(CGCD)HD and (HB)CB(CGCDCE)HE experiments (Yamazaki et al., 1993). Approximately 94.8% of the backbone and 84.6% of side-chain resonances were assigned.

Distance restraints for the structure calculation were obtained from 3D NOESY–^13^C-HSQC and NOESY–^15^N-HSQC spectra (mixing time = 150 ms) (Fesik and Zuiderweg, 1988; Marion et al., 1989).

All 3D NMR spectra were recorded with non-uniform sampling (NUS) to maximize resolution in the respective indirect dimensions. NUS schedules were generated with Poisson-Gap sampling using the on-line Schedule Generator from the Wagner group (http://gwagner.med.harvard.edu/intranet/hmsIST/gensched_new.html). The time-domain data matrices were reconstructed using the hmsIST software with the assistance of the GNU program “parallel” (Tange, 2011; Hyberts et al., 2012). Processing of re-constructed data-sets was done with NMRPipe (Delaglio et al., 1995); processing of uniformly sampled spectra was done with either NMRPipe or Topspin 3.2 (Bruker). All spectra were analysed and assigned using CcpNmr Analysis v2.4 (Vranken et al., 2005).

Backbone dihedral angles (φ, ψ) were calculated with TALOS-N from the backbone chemical shifts (Shen and Bax, 2015). The Chemical Shift Index (Wishart and Sykes, 1994) was calculated with CcpNmr (Supplementary Figure S1).

For structure calculation, the 3D ^15^N- and ^13^C-edited NOESY spectra were analysed in CcpNmr and the intensities of cross-peaks were used to quantify the inter-proton distances. In the NOESY spectra we picked a total of 3,146 peaks, of which we could assign 3,106. Only manually assigned peaks were used in the structure calculations by ARIA 2.3/CNS1.2 (Linge et al., 2003), because the automated peak assignment routine of Aria failed due to severe spectral overlap in the more unstructured protein regions. Unassigned peaks were discarded. In the end, a total of 2,163 unambiguously assigned distance restraints entered structure calculation. We calculated a total of 100 structures, of which the 20 lowest-energy structures were refined in explicit water.

## 3 Results

### 3.1 PYM binds to the SOLE RNA and other RNAs with no apparent specificity

To determine whether the influence of PYM on localization of *oskar* is related to the SOLE RNA, we tested the binding of PYM to the 32mer SOLE RNA (Figure 1A) by NMR. As a PYM construct, we chose to use PYM^1–160^ (Figure 1B), which misses a stretch of 51 amino acids at the C-terminus compared to the full-length protein. PYM^1–160^ has been found sufficient to recapitulate the effect of PYM over-expression on RNA localization (Ghosh et al., 2014); in addition, this construct was considerably more stable than the full-length PYM, which yielded NMR spectra of poor quality and showed significant degradation after 24 h. The chemical-shift perturbations (CSPs) observed in the ^15^N-HSQC spectra of 120 μM ^15^N-labelled PYM^1–160^ upon addition of the 32mer SOLE RNA demonstrated that the protein binds to the RNA (Figure 1D); furthermore, addition of more than 1 molar equivalent of RNA did not cause additional changes in either the intensity or the position of the NMR peaks.

To find out whether PYM binds specifically to the SOLE RNA and if so, which of its structural elements are responsible for the binding, we designed several other RNAs and tested their interactions with PYM^1–160^ by electrophoretic mobility shift assays (EMSAs). The RNAs tested consisted of : 1) the SOLE RNA PS capped by a tetraloop (22mer PS RNA); 2) SOLE RNA constructs with progressively shortened PS stem by removal of nucleotides at 5′ and 3′ ends to yield 26mer, 24mer, 22mer (which corresponded to the 20mer SOLE RNA sequence with the addition of a terminal G–C base pair), 20mer and 16mer RNAs; 3) two 12mer single-stranded (ss) RNAs with random sequences (ssRNA_1 and ssRNA_2); 4) a double-stranded (ds) RNA of 11 canonical base pairs (dsRNA_1); 5) a dsRNA of 9 base pairs, including one non-canonical G–A and one non-canonical G–U base pair (dsRNA_2).

In the EMSAs, we evaluated the disappearance of the band corresponding to the free RNA upon titration of increasing molar equivalents of the protein. We were unable to see a clear band corresponding to the PYM^1–160^–RNA complex, both because the strong positive charge of the protein (pI = 9.7) impaired the penetration of the complexes into the gel, and because the fast dissociation rate of some of the complexes (for example, that with the 32mer SOLE RNA) led to a smeared band. Because of the lack of a clearly-defined bound-RNA band, we refrained from quantification of the EMSA data. However, PYM^1–160^ shifted all RNAs, demonstrating that the SOLE RNA is not a specific target of PYM (Figure 1C).

### 3.2 PYM^1–160^ is an intrinsically disordered protein

**FIGURE 2:**
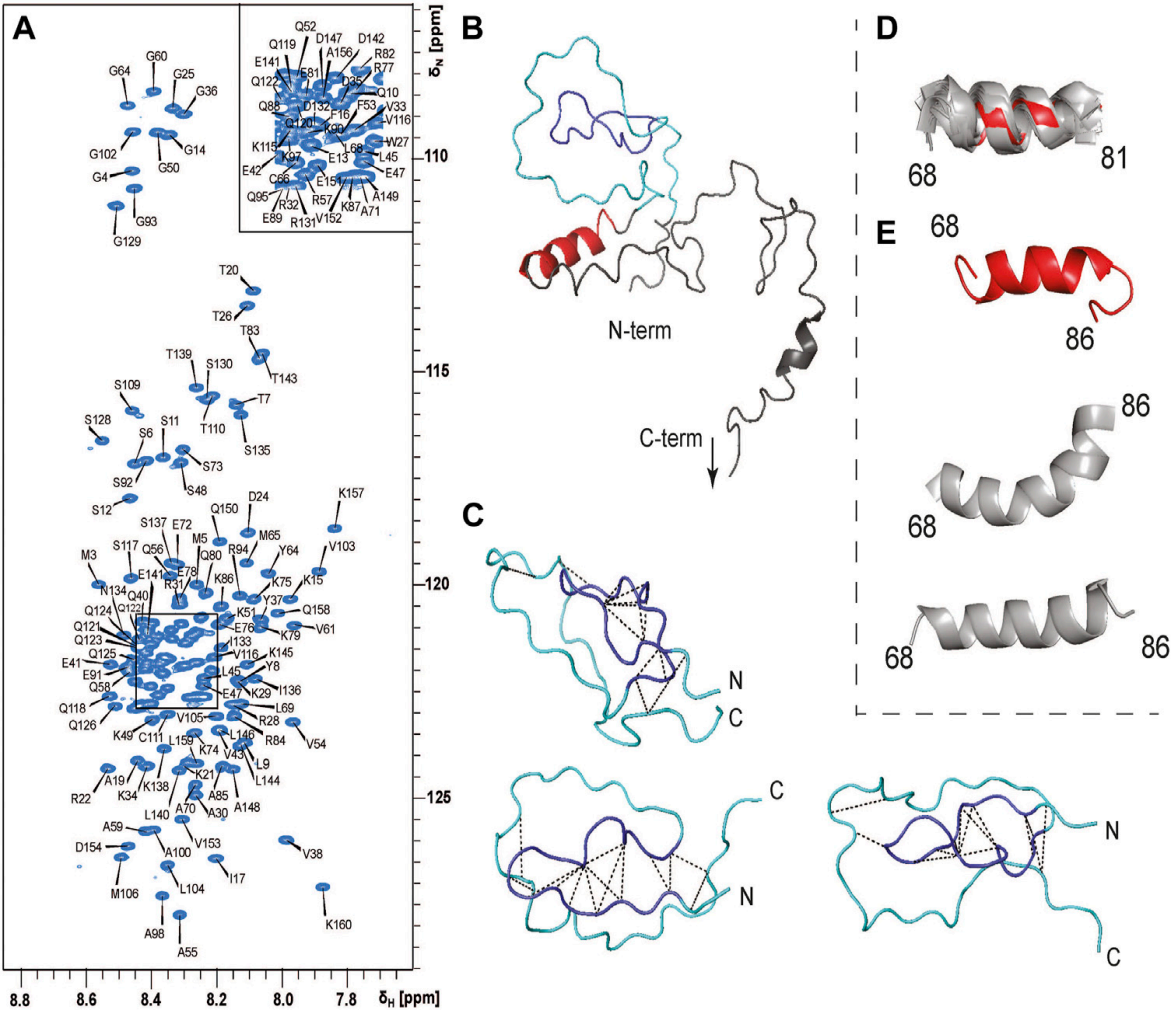
PYM^1–160^ is an intrinsically unfolded protein. **(A)** ^15^N-HSQC spectrum of PYM^1–160^ with peak assignments. The limited dispersion of the H^N^ chemical shifts is indicative of a largely unfolded protein. **(B)** The lowest energy structure of PYM^1–160^. The β-domain, emulating a β-strand–turn–β-strand motif, is in blue (aa 16–33), the region comprising stretches 9–16 and 34–65, where long-range NOEs are observed, is in cyan, and the helix 68–81 is in red. The most C-terminal stretch is not shown, as it is completely disordered. **(C)** Three representative structures of the region 13–65, with the detected long-range NOEs shown as black dashed lines. **(D)** Overlay of helix 68–81 in the 20 lowest-energy structures. The lowest energy structure is in red and all other structures are in grey. **(E)** Three representative conformations of the stretch 68–86 (the lowest-energy structure is in red, the other structures are in grey).

To understand which regions of PYM^1–160^ are involved in RNA recognition, we assigned the NMR signals of PYM^1–160^ in solution (Figure 2A). The narrow ^1^H chemical-shift dispersion of the peaks in the ^15^N-HSQC spectrum revealed that the protein is mostly unfolded. Nevertheless, we completed the ^1^H, ^13^C and ^15^N backbone and side-chain resonance assignments for PYM^1–160^ and subsequently the near-complete peak assignment of 3D ^15^N- and ^13^C-edited NOESY spectra. The Chemical Shift Index and the NOE analysis indicated the presence of an α-helix between amino-acids (aa) 68 and 86 with a possible discontinuity at aa 83 (Supplementary Figure S1). A few additional helical turns are predicted in the range 115–160. Despite the absence of a well-defined secondary structure, the N-terminal region of the sequence (aa 15–35) showed a few long-range NOEs between aa 16–17 and 31–33 (Figure 2C), which are compatible with the structure of PYM^3–35^ in complex with the Mago–Y14 dimer solved by crystallography (PDB entry: 1rk8). We conclude that PYM^15–35^ transiently forms a strand-turn-strand β-domain. To confirm this, we calculated the structure of PYM^1–160^ using ARIA 2.3/CNS1.2 (Figure 2B). We detected the formation of a loosely defined β-sheet-type structure in the range 16–33, which approximates a two-stranded β-sheet with connecting β-hairpin (root-mean-squared-deviation, RMSD, of backbone atoms in the range 16–33 with respect to the lowest-energy structure = 3.4 Å). Additional long-range NOEs in the range 9–65 (Figure 2) indicate the formation of a loop in the stretch 35–60, but the relative position of the two-stranded β-sheet structure and the loop formed by the 35–60 stretch varies among the 20 lowest energy structures (Figure 2C; Supplementary Materials). The NMR structural ensemble (Supplementary Materials) clearly reveals the tendency to form secondary and tertiary structures in the range 9–65. These structures represent only a subset of the conformational space sampled by this region of the protein. The determination of the full conformational space sampled by PYM requires fitting the experimental data to a large pool of co-existing structures and is outside the scope of this work.

Finally, the structure calculations confirmed the presence of a stable helix extending from aa 68 to 81 (Figure 2D), which was found in all 20 lowest-energy structures (backbone RMSD over residues 68–81 with respect to the lowest-energy structure = 1.2 Å). In some structures, the helix extended until aa 86, forming either a continuous bent helix from aa 68 to 86, or a helix-turn-helix structure (Figure 2E).

In conclusion, PYM is a predominantly disordered protein with some short secondary-structure elements and a few tertiary contacts in the N-terminal domain.

### 3.3 PYM^1–160^ in complex with RNA

**FIGURE 3:**
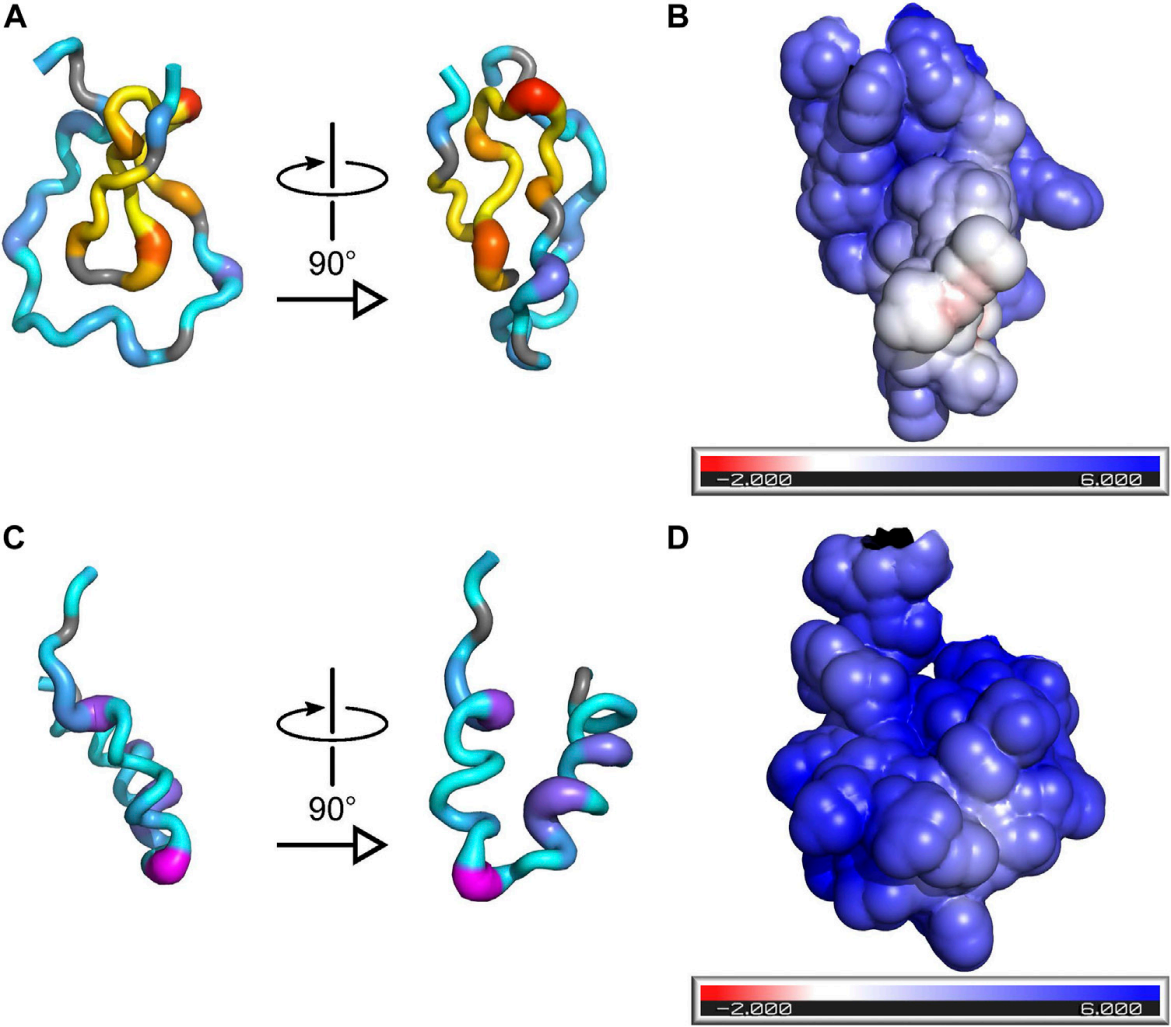
Binding of RNA to PYM is incompatible with formation of the PYM–Mago–Y14 complex. **(A)** Two orthogonal views of a cartoon representation of PYM^15–65^ with color and thickness set according to the magnitude of the CSPs induced by binding of the 32mer SOLE RNA. The color-scheme for residues 18–39, which are involved in recognition of the Mago–Y14 heterodimer in the structure of PDB ID 1rk8, is yellow-to-red (low-to-high CSP); the color-scheme for all other residues is cyan-to-magenta. **(B)** Surface representation of PYM^15–65^ showing the solvent-accessible surface colored according to the electrostatic potential (red: negative potential; blue: positive potential). The orientation of the protein is the same as in the right-hand view of **(A)**. **(C)** As **(A)** but for PYM^66–98^. **(D)** As **(B)** but for PYM^66–98^; the orientation of the protein is the same as in the right-hand view of **(C)**.

The ^1^H chemical-shift dispersion of the peaks in the ^15^N-HSQC spectrum of PYM^1–160^ remains narrow in the presence of RNA, demonstrating that the protein does not fold into a well-defined tertiary structure in complex with RNA (Figure 1D). To gain information on the regions of the protein involved in RNA interactions, we measured chemical-shift perturbations (CSPs) for 30 μM PYM^1–160^ in the presence of 0.5, 1 and 2 molar equivalents of RNA. We used a subset of the RNA constructs analysed by EMSA: the 32mer, 26mer and 20mer SOLE RNAs, the 22mer PS RNA, dsRNA_1 and ssRNA_2 (Supplementary Figure S2). One molar equivalent of 32mer SOLE RNA generated CSPs above average in the protein region 17–94. In agreement with the biological data (Ghosh et al., 2014), which attributes PYM function in *oskar* localization predominantly to the N-terminal part of the protein, we did not detect any contacts between any RNA and PYM^1–160^ beyond residue 100. A positively charged surface formed by the pseudo-globular domain 17–65 accounts for some of the largest CSPs due to RNA binding (I17, R22, T26, A30, K34, V38 and E41; Figure 3). These residues are in the same region of the protein shown to interact with the Mago–Y14 complex (Supplementary Figure S3) (Bono et al., 2004). Additional large chemical-shift perturbations were observed for V54, A55 and A59, in the helix 68–86 (K74, E78, K79, Q80 and T83) and the adjacent residues S92 and G93 (Figure 3; Supplementary Figure S2).

The patterns of the CSPs produced by the different RNAs were all similar, demonstrating that the same protein residues are involved in interaction with all RNAs, independently of their sequences or structures. However, while the magnitudes of the protein CSPs at an RNA:protein ratio of 1:1 produced by the 26mer and 20mer SOLE RNAs as well as the 22mer PS RNA and dsRNA_1 were only slightly smaller than those caused by the 32mer SOLE RNA, 1 molar equivalent of ssRNA_2 produced much smaller CSPs, which, at two equivalents, increased to half the size of those caused by the 32mer SOLE RNA (Supplementary Figure S2).

**FIGURE 4:**
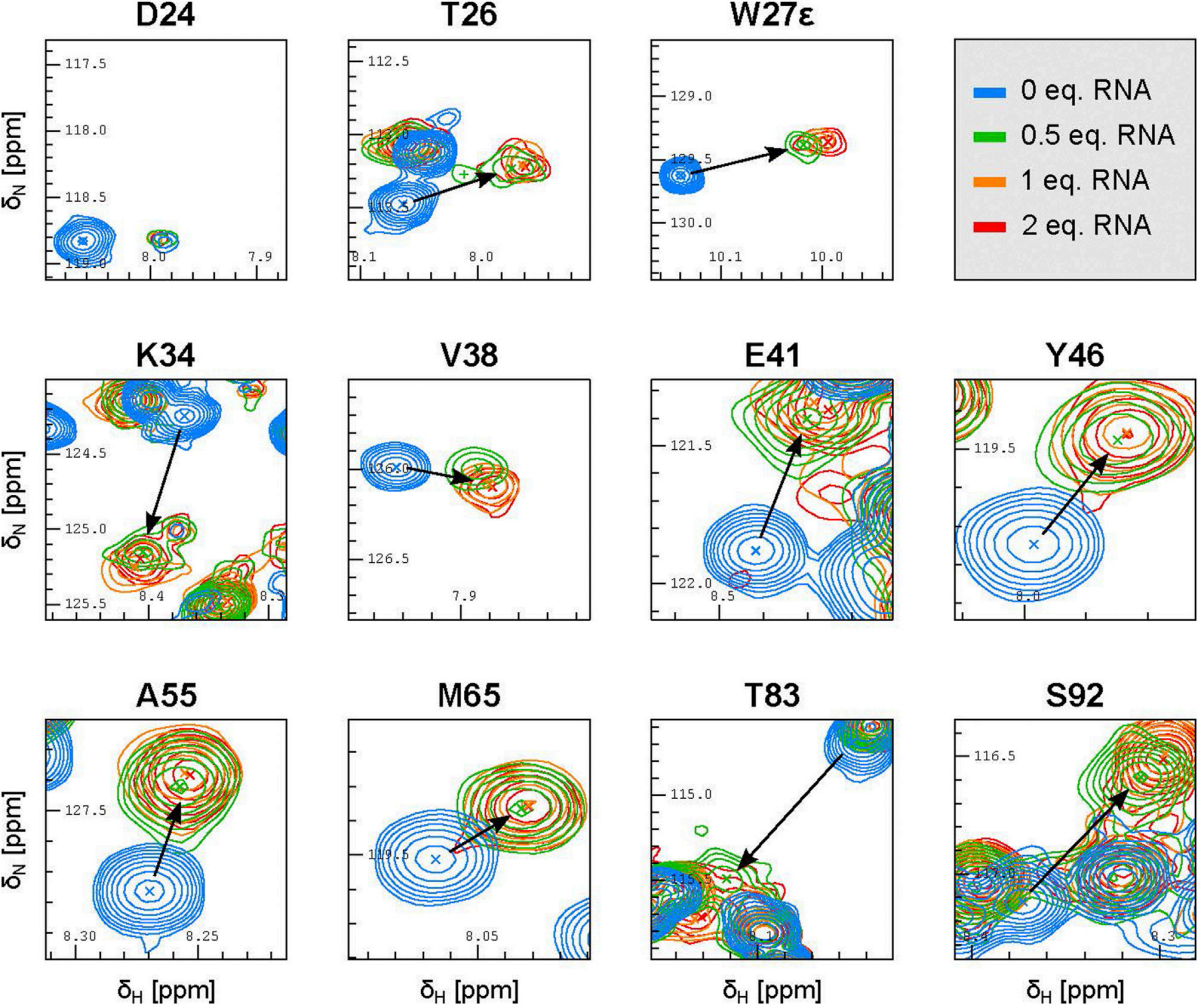
The SOLE RNA binds to PYM with a 1:2 RNA:protein stoichiometry. Excerpts of representative peaks in ^15^N-HSQC spectra of 30 μM PYM^1–160^ in the presence of 0.5 (green), one (orange), two (red) equivalents of the 32mer SOLE RNA.

Careful inspection of the ^15^N-HSQC spectra where the RNA was added in 0.5, 1 and 2 molar equivalents with respect to PYM^1–160^ (at 30 μM) revealed additional features of PYM RNA recognition. At 0.5 equivalents of 32mer SOLE RNA, the peaks of PYM^1–160^ were shifted nearly all the way to their final position (Figure 4; Supplementary Figure S4). At one RNA equivalent, the peaks moved slightly further and reached their final position. This data is consistent with the formation of a PYM_2_–RNA complex rather than a 1:1 complex, with *K*_D_ less than 1 μM^2^. Inspection of the same spectra of PYM^1–160^ upon addition of 0.5 equivalents of either the 26mer SOLE RNA, the 22mer PS RNA or dsRNA_1 revealed two distinct RNA-bound species with very different intensities. The major (more-intense) peaks behaved as for the 32mer SOLE RNA while the minor (less-intense) peaks corresponding to the second species were weak and not always detectable. Assuming that PYM^1–160^ exchanges rapidly between each of the RNA-bound states and the unbound state, but that the two RNA-bound states exchange only slowly (or not at all) with each other (Eqs 1, 2), the major and minor peaks appearing at 0.5 equivalents of the 26mer SOLE RNA, 22mer PS RNA or dsRNA_1 can be interpreted as corresponding to PYM_2_–RNA and PYM–RNA_x_ complexes, respectively (or more rigorously, to the fast-exchanging mixtures of these two complexes with the free protein). Because of the weak intensities of the minor peaks, we were unable to determine the value(s) of x. Only with the RNA constructs shorter than the 32mer SOLE RNA could the peaks corresponding to the PYM–RNA_x_ species be clearly detected at 0.5 equivalents of RNA (Figure 4; Supplementary Figure S4). The assumption that PYM^1–160^ exchanges rapidly between the RNA-bound and free states in the PYM_2_–RNA complex was verified in titration experiments with the 32mer SOLE RNA using 0.1, 0.2, 0.3 and 0.5 equivalents of RNA (Supplementary Figure S5).

$$PYM+x∙RNA\overset{K_{D,1}}{\to \leftarrow }PYM–{RNA}_{x}$$ (1)

$$2\cdot PYM+RNA\overset{K_{D,2}}{\to \leftarrow }{PYM}_{2}–RNA$$ (2)

The 32mer and 26mer SOLE-RNAs, as well as the 22mer PS RNA and dsRNA_1, contain a helical structure of at least 9 base pairs, which approximately corresponds to one helical turn. From our NMR spectra, we conclude that this secondary-structure element favours the formation of an RNA–protein complex with a 1:2 stoichiometry. However, shorter helical constructs, such as the 26mer SOLE RNAs or the 22mer PS RNA, appear to form less stable PYM_2_–RNA species, increasing the relative population of complexes with different stoichiometry. In the presence of ssRNA_2 and of the 20mer SOLE RNA, which is predicted to form an unstable double-stranded structure, the relative intensity of the minor peak with respect to the major peak is greater than in the presence of the double-stranded RNAs. This suggests that, while the same residues are involved in RNA binding, the average stoichiometry of binding may differ between double-stranded and single-stranded RNA.

When we compared the amount of unbound RNA detected in EMSA assays with the size of the chemical-shift perturbations across the different RNA constructs, we observed that the values do not correlate. For example, while in the NMR experiments, double-stranded RNA constructs, such the 22mer PS RNA and dsRNA_1, are nearly fully bound to PYM^1–160^ at an RNA:protein ratio of 1:1, the EMSA assays show that 60% and 50% of the 22mer PS RNA and dsRNA_1, respectively, are still unbound at this RNA:protein ratio, and 40% and 20% of the RNAs remain unbound at an RNA:protein ratio of 1:5. Because the EMSA assays used RNA at 5 μM, only 6-times less concentrated than the protein in NMR experiments, these discrepancies are too large to be explained by the differences in the concentration of the components in the two assays. EMSA is a non-equilibrium assay that uses RNAs coupled to a bulky fluorescent tag at one end; thus, it is possible that different factors (i.e., the gel matrix, running buffer, aberrant 5′-end effects, etc.) impact the intensity of the unbound RNA band (Hellman and Fried, 2007). Because we used EMSA merely to prove that PYM does not specifically bind the SOLE RNA rather than to extract binding constants, we did not further optimize the EMSA conditions and turned to NMR to characterize the mode of binding.

Finally, a construct of the 32mer SOLE RNA with a mutated MSL incapable of forming base pairs bound PYM^1–160^ identically to the 32mer wild-type SOLE RNA (Supplementary Figure S6), indicating that the nature of this structural element is irrelevant for binding.

## 4 Discussion

Our work reveals that PYM^1–160^ is an intrinsically unfolded protein with the exception of an α-helical region comprising aa 68–81. In the structure of PYM^1–58^ bound to the EJC proteins Y14 and Mago, the first 35 residues form a three-stranded β-sheet and a contiguous β-hairpin (Supplementary Figure S3) (Bono et al., 2004). While we do not see this structure in free PYM, the region comprising aa 16–33 emulates a loosely formed β-strand–turn–β-strand motif, and may be considered as a folding intermediate of the structure seen in complex with the Mago–Y14 dimer. The presence of a number of long-range NOEs in the region 13–65 clearly indicates that the N-terminal part of PYM^1–160^, despite not being properly folded, populates only a sub-space of the fully unfolded conformational landscape and shows a significant preference for more compact, globular-like conformations.

The formation of a globular-like region results in a co-localization of positive charges that build an RNA-binding surface. Binding to RNA does not induce folding of PYM into a rigid structure but instead largely preserves its disordered character, thereby maintaining the conformational entropy of the protein in the complex. PYM engages amino acids in the stretch 22–34 in RNA binding. These residues, such as the positively charged side-chains of R22, R28, K29, R31 and K34, are also involved in contacts with the Mago–Y14 dimer (Supplementary Figure S3) (Bono et al., 2004), suggesting that the two interactions are mutually exclusive. The extensive plasticity of the first 65 amino acids of PYM may be required to allow independent molecular recognition by both RNA and protein binding partners.

The RNA binding properties of *Dm* PYM were first reported in (Bono et al., 2004). However, in this paper, the binding of RNA was found to be compatible with that of Mago–Y14 by band shift assays. Our data challenge this finding. As the RNA used in the band shift assays of (Bono et al., 2004) is longer than the RNAs used in our work, it is conceivable that the binding of Mago–Y14 to the PYM–RNA complex occurs on a composite PYM–RNA surface that does not include the surface of PYM proven to interact with the Mago–Y14 complex in the absence of RNA. This complex could potentially be an intermediate in the process of handing over PYM from the RNA to the Mago–Y14 heterodimer during EJC recycling.

The EJC is essential for localization, as demonstrated by (Ghosh et al., 2012). In the presence of PYM, the EJC can be dissociated and recycled, a process which—if dysregulated—interferes with the localization of *oskar* mRNA. The formation of the PYM–RNA complex may serve the purpose of preventing PYM from acting on the EJC before mRNA localization has been accomplished. Whether the SOLE RNA participates in this process remains to be determined, since our studies suggest that PYM^1–160^ does not bind the SOLE RNA specifically, at least *in vitro*. However, the PS element, which is essential for localization (Ghosh et al., 2012), appears to enhance the stability of the PYM_2_–RNA complex relative to complexes with different stoichiometry. Similarly, the sequence of the MS element, which is irrelevant for localization (Ghosh et al., 2012), also appears to be unimportant for binding to PYM (Supplementary Figure S6). Thus, the same structural features of the SOLE RNA that are necessary for localization are also required for optimal PYM binding. While this does not demonstrate that PYM binds the SOLE RNA *in vivo*, it links the role of the SOLE RNA in localization with its PYM recognition features, making a role of the SOLE RNA in both mRNA localization and PYM binding at least plausible. The lack of sequence specificity in RNA recognition by PYM may be dictated by the function of PYM in different cellular contexts. The proposed role of the mRNA in down-regulating PYM activity before it reaches the target location is endorsed by the finding that over-expression of PYM inhibits *oskar* mRNA localization in a manner that is dependent on the relative amount of PYM and mRNA (Ghosh et al., 2014).

Interestingly, the RNA binding surface of PYM in the globular-like domain is conserved from flies to humans (Supplementary Figure S7), suggesting a similar role for this PYM structural region across organisms. PYM has been shown to interact with the Mago–Y14 heterodimer in both fly oocytes and human cell lines; however, while in *D. melanogaster*, PYM acts to facilitate EJC recycling in mRNA localization, in humans PYM interacts with the ribosome, where it facilitates mRNA translation by stripping off the EJC (Gehring et al., 2009; Ghosh et al., 2014). The C-terminal domain of PYM was found to be required for ribosome binding in human cells (Gehring et al., 2009), but the conservation of PYM sequence in this region does not account for the inability of PYM to bind to the ribosome in flies. The region of least homology between fly and human PYM is in the amino acid stretch found to form an α-helix in *Dm* PYM, with the human protein featuring an insertion of 8 amino acids as well as several proline and glycine residues. Our data show that this region of *Dm* PYM, and the disordered stretch following it, are involved in RNA binding. Whether the divergent sequences in this region, and potentially, their different RNA binding properties, recapitulate the different functions of PYM in the two organisms, remains to be determined.

Using a combination of NMR spectroscopy and EMSAs we have demonstrated that RNA helical elements recognize the unfolded PYM protein in a manner that is principally dependent on the overall structural fold of the RNA rather than the nucleotide sequence, and which does not result in well-structured protein–RNA interfaces. The driving force for the interaction is charge complementarity, together with preservation of conformational entropy. Notably, such interactions have been previously observed between two intrinsically unfolded proteins or unfolded protein stretches and have been associated with specific cellular functions (Borgia et al., 2018; Danilenko et al., 2019). We suggest that functional, fuzzy interactions between charged molecules may be much more common than previously thought, with the less well-defined energy landscape underlying such interactions allowing the participants to fulfill multiple roles in different cellular pathways.

## Data Availability

The original contributions presented in the study are included in the article/Supplementary Material, further inquiries can be directed to the corresponding author.
