# Supplementary material for: The EJC disassembly factor PYM is an intrinsically disordered protein and forms a fuzzy complex with RNA
Source: Front Mol Biosci. 2023 Mar 30;10:1148653. doi: 10.3389/fmolb.2023.1148653 (PMC10098021; doi:10.3389/fmolb.2023.1148653)
Supplement: Supplementary file 1 [file Table1.docx]

Supplementary Material

**The EJC disassembly factor PYM is an intrinsically disordered protein and forms a fuzzy complex with RNA.**

Deepshikha Verma^1^, Veena Hegde^1^, John Kirkpatrick^2^, Teresa Carlomagno^2*^

^1^ Laboratory of NMR-based Integrative Structural Biology, Centre for Biomolecular Drug Research (BMWZ) and Institute of Organic Chemistry, Leibniz University Hannover, Schneiderberg 38, 30167 Hannover, Germany

^2^ Laboratory of Integrative Structural Biology, School of Biosciences, College of LES, University of Birmingham, Edgbaston B15 2TT, Birmingham, United Kingdom

*** Correspondence:** Teresa Carlomagno: [t.carlomagno@bham.ac.uk](mailto:t.carlomagno@bham.ac.uk)

# Supplementary Figures

**
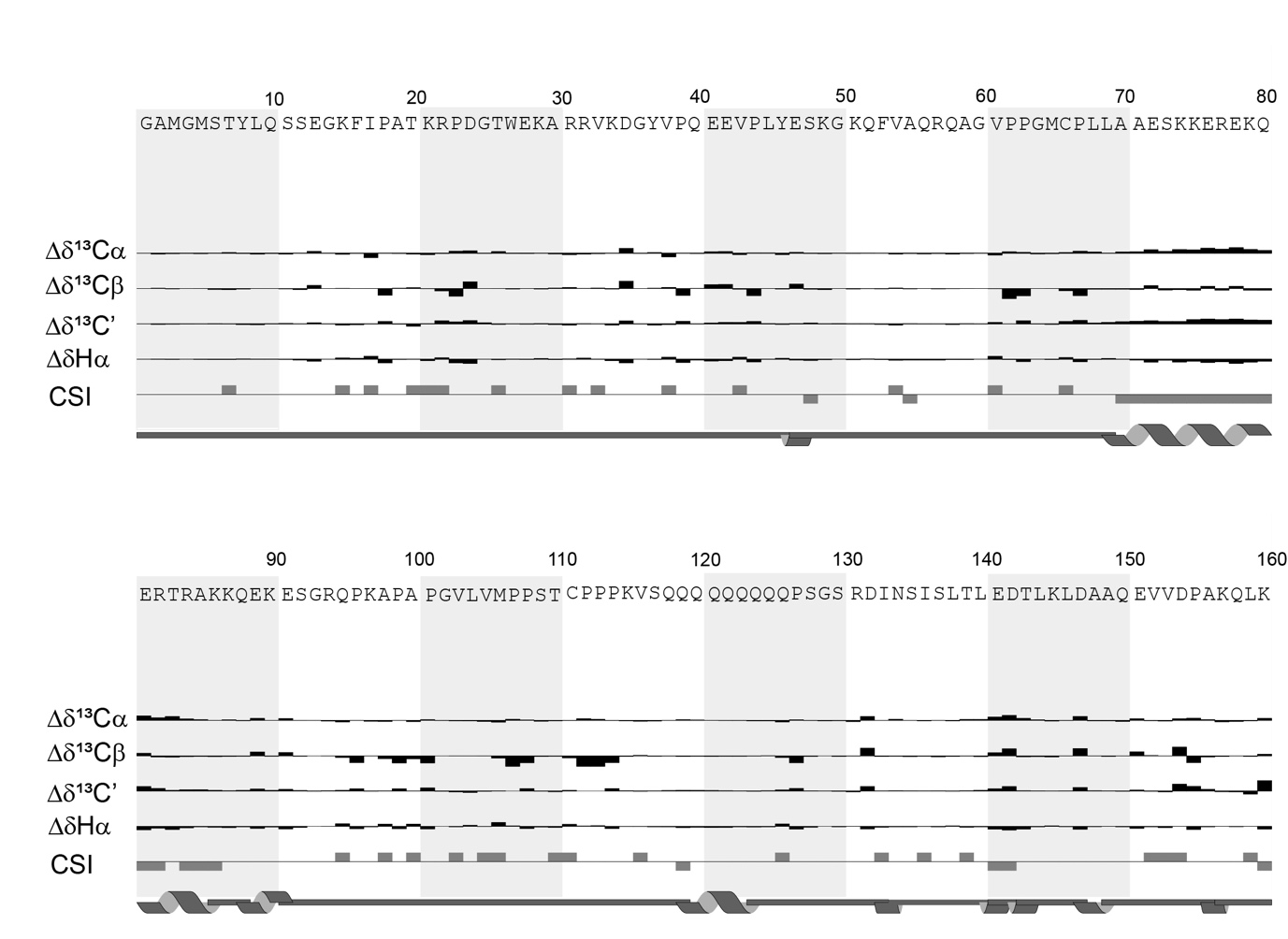
**

**Supplementary Figure 1.** **Analysis of the secondary structure of PYM^1–160^ based on NMR chemical shifts and NOEs**. The first four lines represent the deviation of the chemical shifts measured for the corresponding position along the protein sequence (shown at the top) from the chemical shifts of the same amino-acid type in random coil regions, shown on a relative scale (Δδ); the fifth line represents the value of the chemical shift index (CSI) (Wishart and Sykes, 1994). Below the table are shown the secondary structure elements, as predicted by DANGLE (Cheung et al. 2010 *J Magn Reson* 202, 223).

**
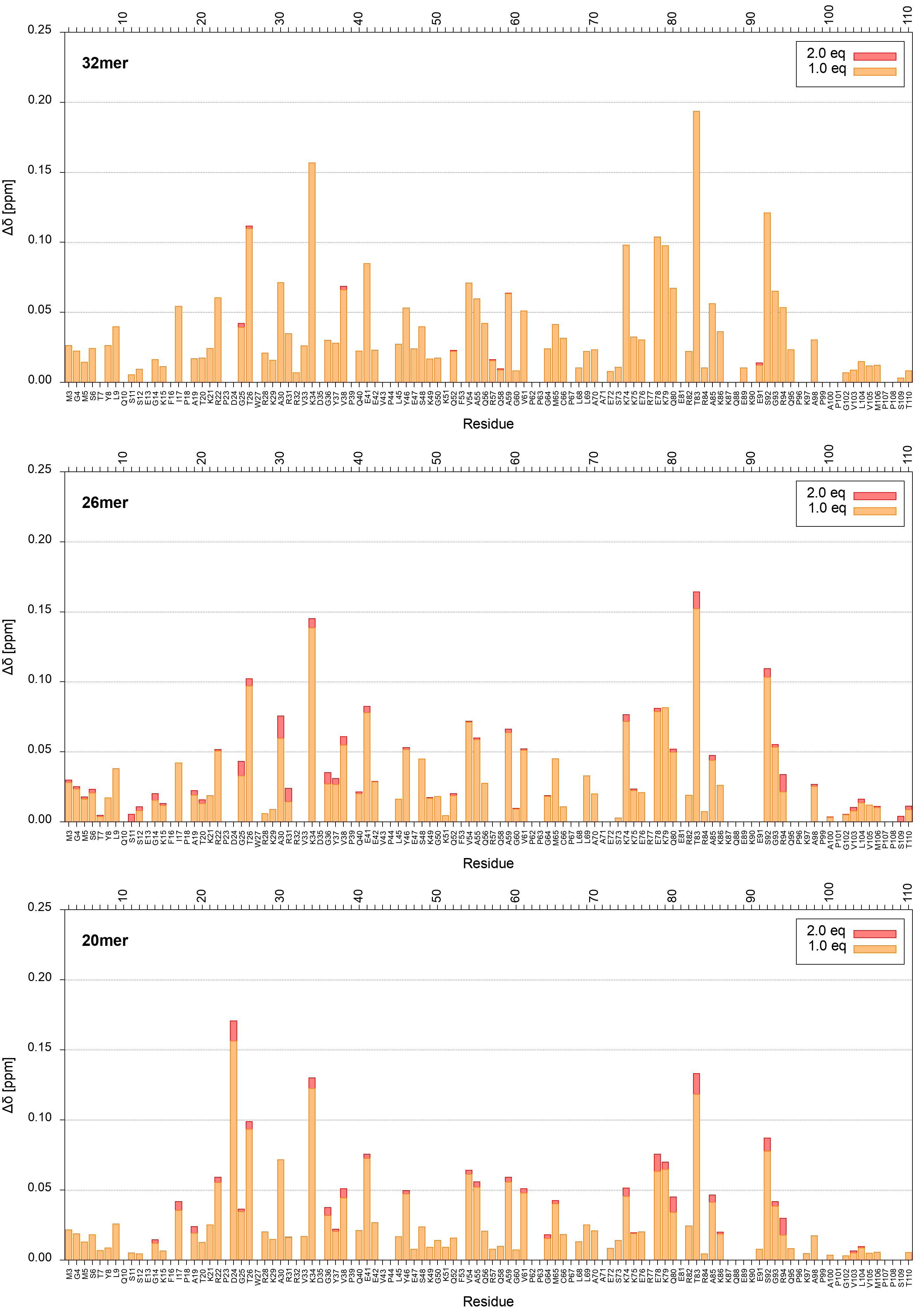
**

**
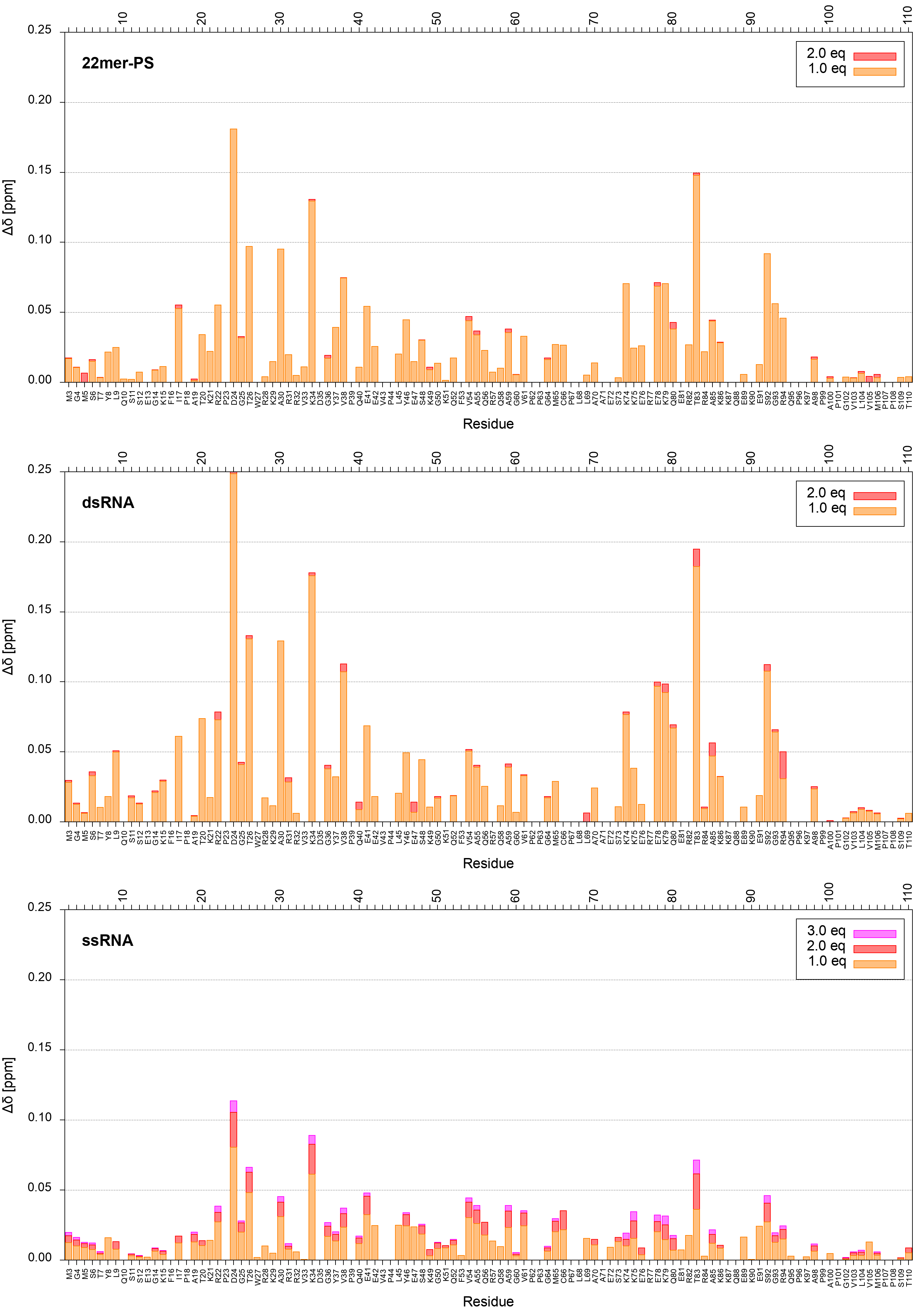
Supplementary Figure 2.** **Plots of chemical-shift perturbations (CSPs) of ^15^N-HSQC peaks of PYM^1–160^ measured in the presence of several RNA constructs.** The protein concentration was 30 μM in all experiments. The RNA:protein ratio was 1:1 (orange), 2:1 (red) or 3:1 (magenta). In the case of the 32mer SOLE RNA, the protein is fully bound to the RNA at an RNA:protein ratio of 1:1. For the shorter double-stranded RNA constructs, such the 26mer and 22mer SOLE RNAs, the 22mer PS RNA and dsRNA_1, PYM^1–160^ is fully bound at an RNA:protein ratio of 2:1. Only for ssRNA_2 did the protein CSPs still increase beyond two equivalents of RNA, and the absolute values of the CSPs at the final titration point (three equivalents of RNA) remained much smaller than those corresponding to full-binding to the RNAs containing double-stranded structures.

**
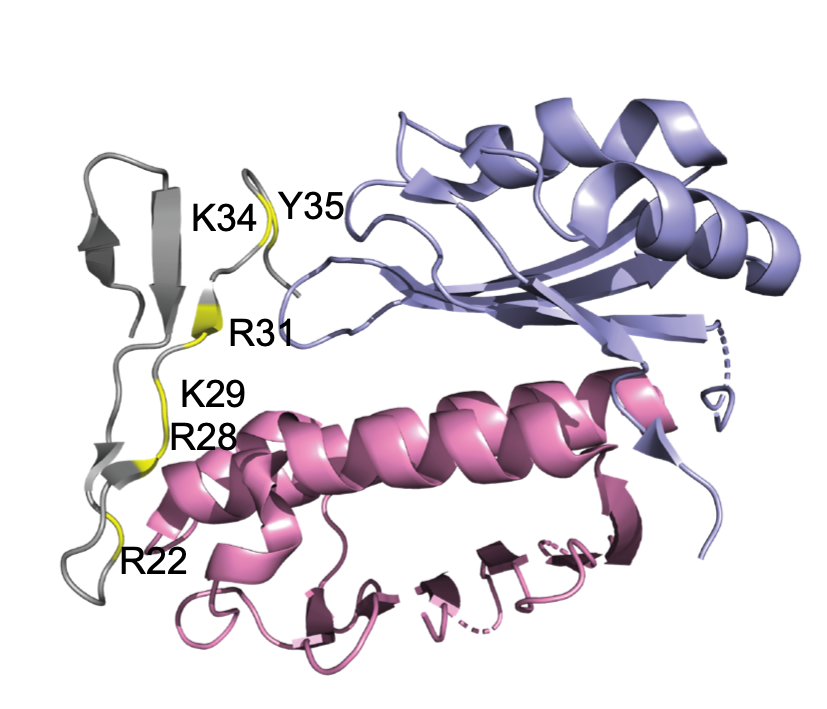
**

**Supplementary Figure 3.** Crystal structure of PYM^1–58^ (grey) bound to Mago (pink) and Y14 (light blue) (PDB ID 1rk8). The residues of PYM in contact with either Mago or Y14 are highlighted in yellow and annotated.

**
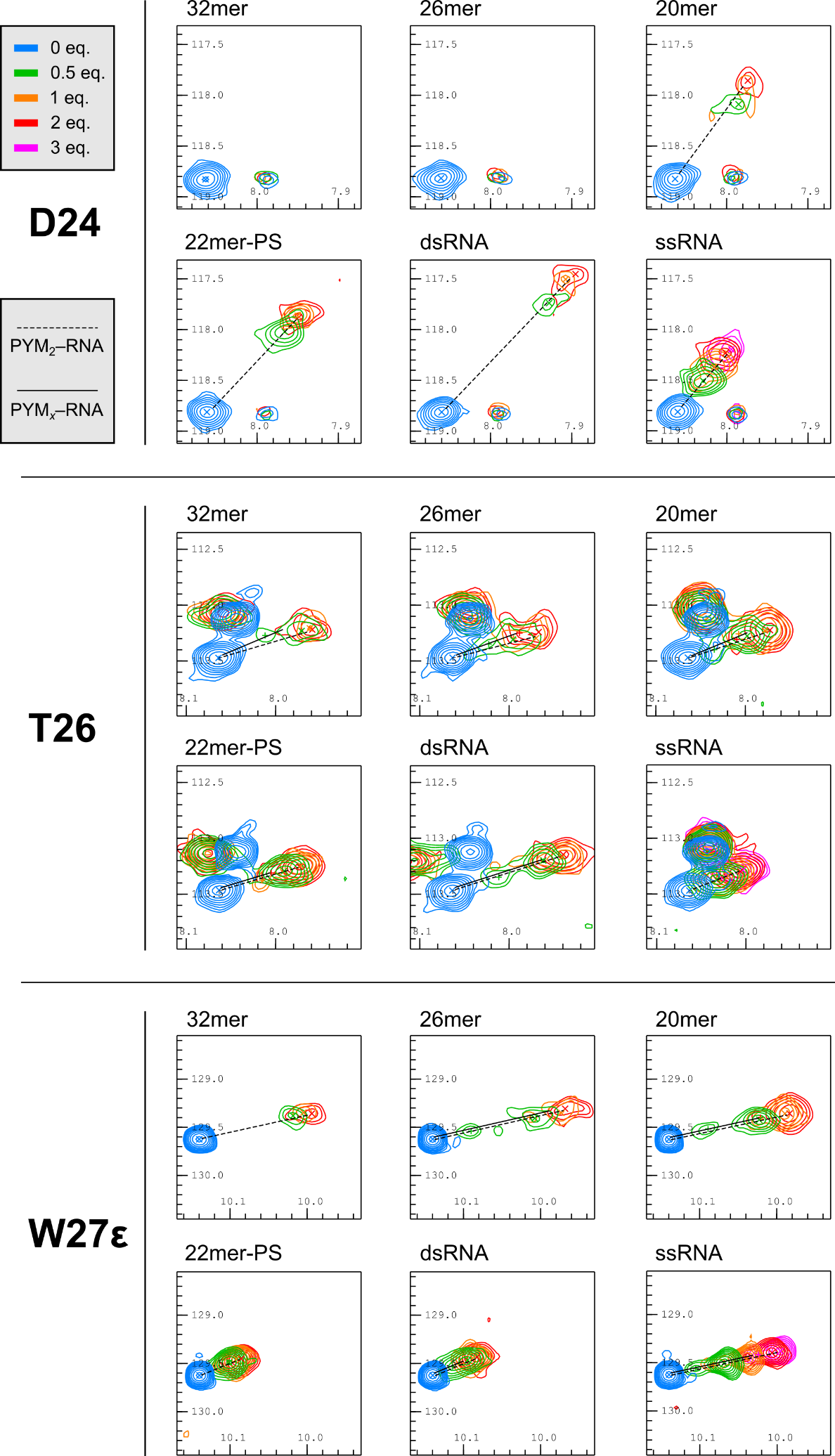
**

**
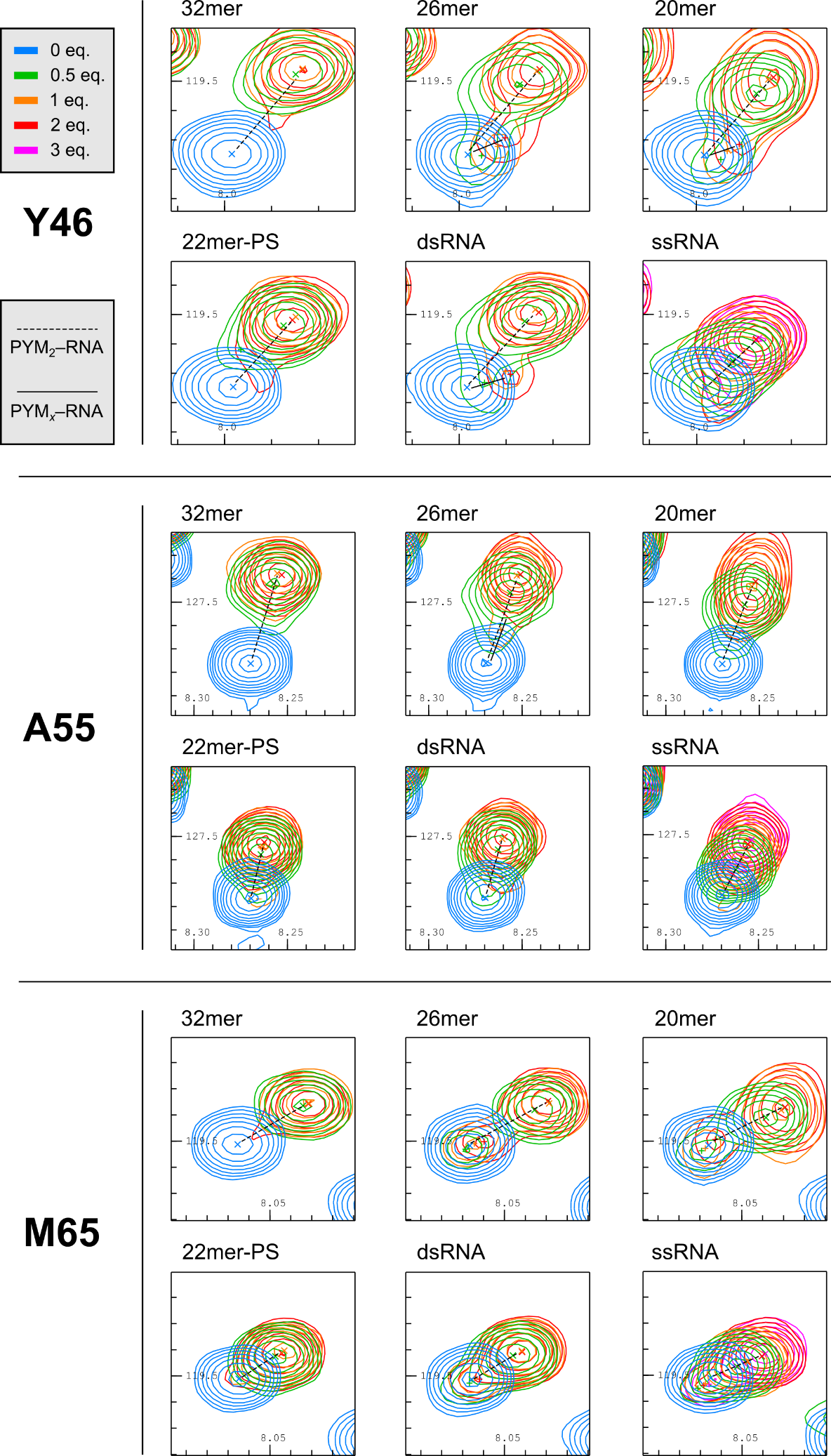
**

**
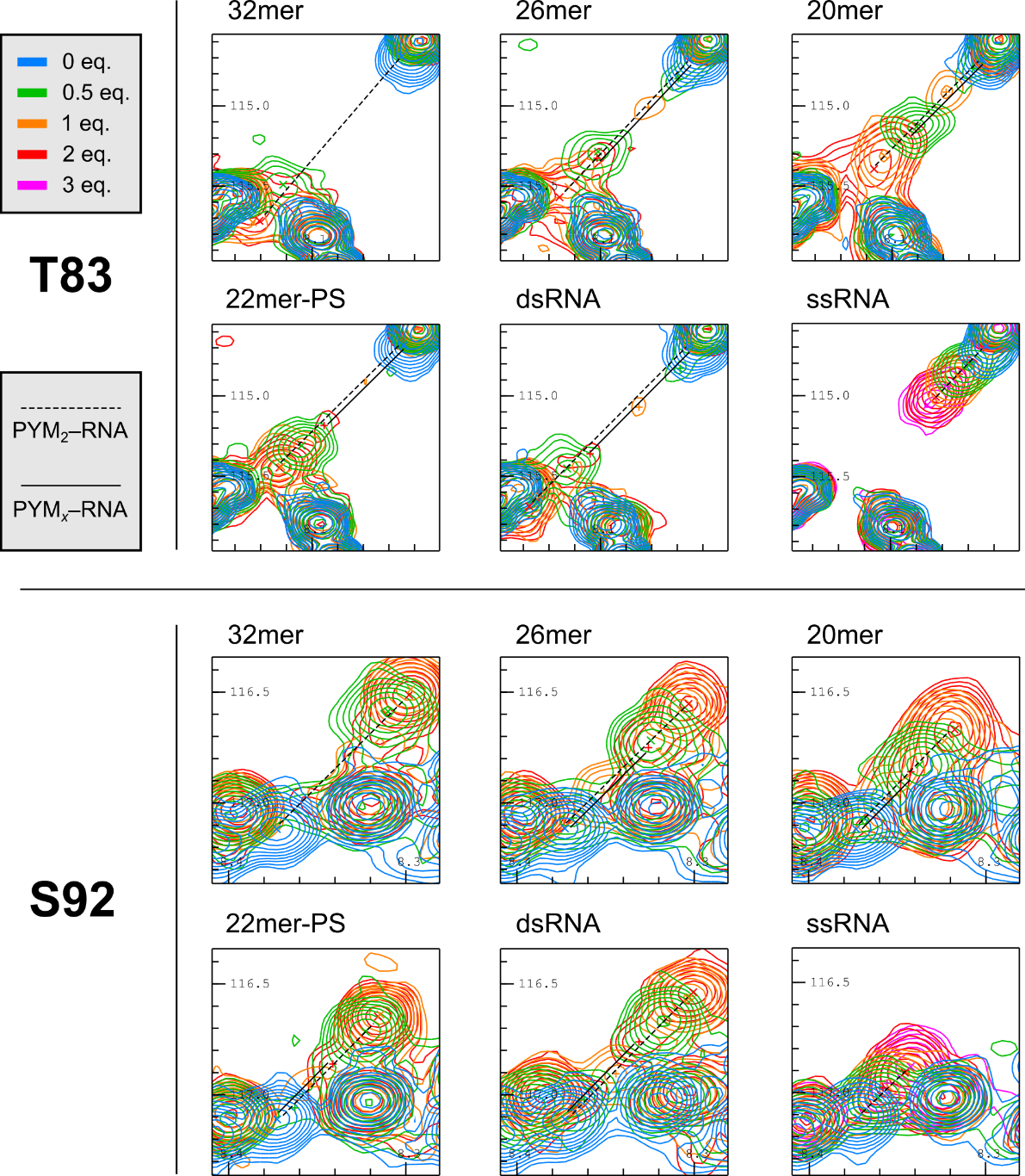
**

**Supplementary Figure 4. RNA binding to PYM includes the formation of two complexes with distinct binding stoichiometry.** Excerpts of representative peaks in the ^15^N-HSQC spectra of 30 μM PYM^1–160^ in isolation (blue), and in the presence of 0.5 (green), one (orange), two (red) and three (magenta) equivalents of the 32mer SOLE RNA (32mer), the 26mer SOLE RNA (26mer), the 20mer SOLE RNA (20mer), the 22mer PS RNA (22mer-PS), dsRNA_1 (dsRNA) and ssRNA_2 (ssRNA). The spectrum after addition of three equivalents of RNA is shown only for the ssRNA_2 construct. The major-peak-trajectories corresponding to the inter-conversion of free PYM with the PYM_2_–RNA complex are indicated with dashed line. Where the minor peaks due to formation of the PYM–RNA_x_ complex(es) are resolvable, the corresponding trajectories are indicated with a solid line. In most cases, the directions of the two trajectories are close to parallel, but deviations are apparent for some residues (e.g. E41 & Y46).


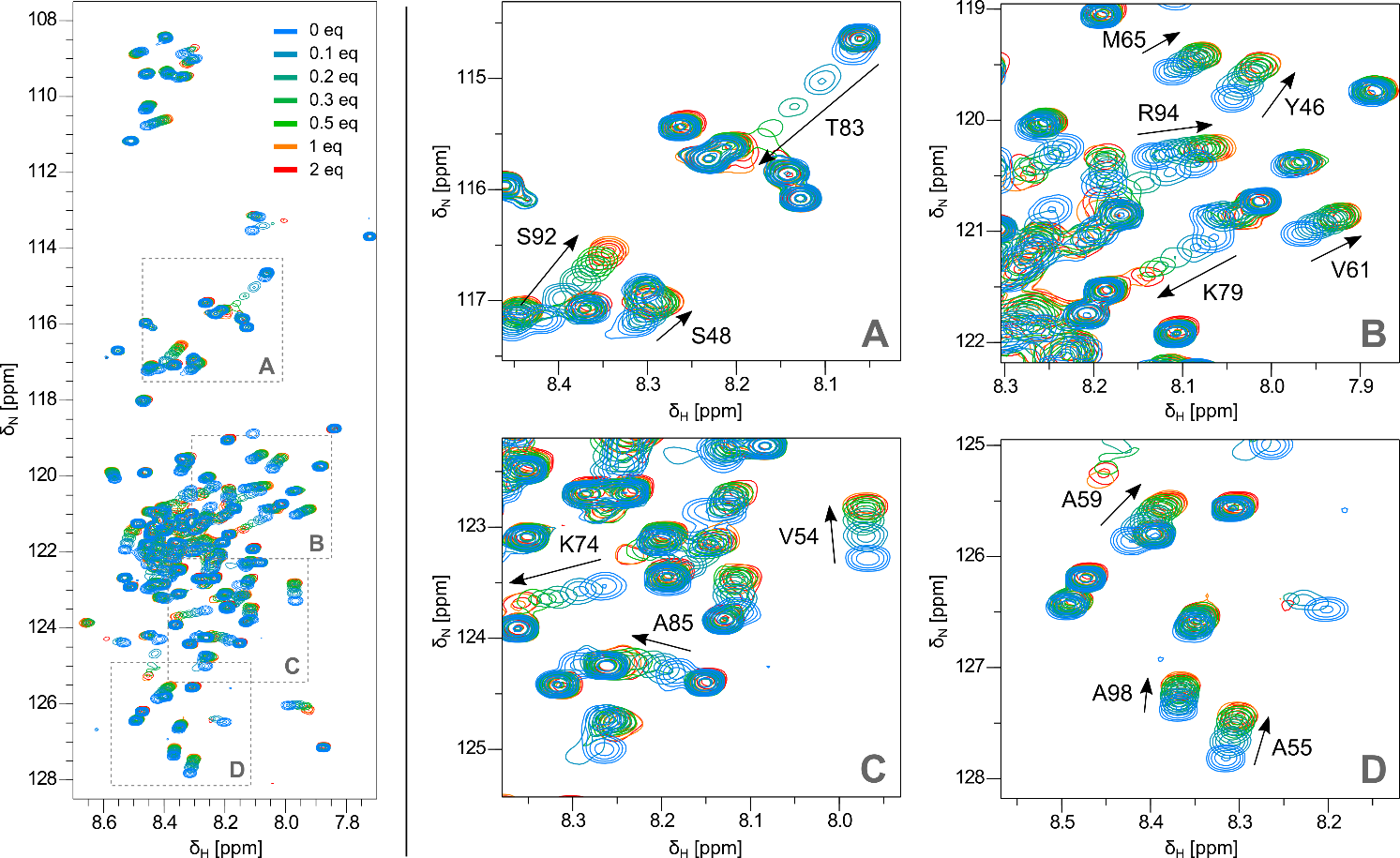


**Supplementary Figure 5.** Overlay of ^15^N-HSQC spectra from the titration of 30 μM PYM^1–160^ with 32mer SOLE RNA, including titration points at 0.1, 0.2 and 0.3 molar equivalents of RNA to illustrate the fast-exchange between free and RNA-bound protein. Left panel: entire amide region. Right panel: expansions of the regions indicated by the dashed boxes (labelled A–D) in the left panel.

**
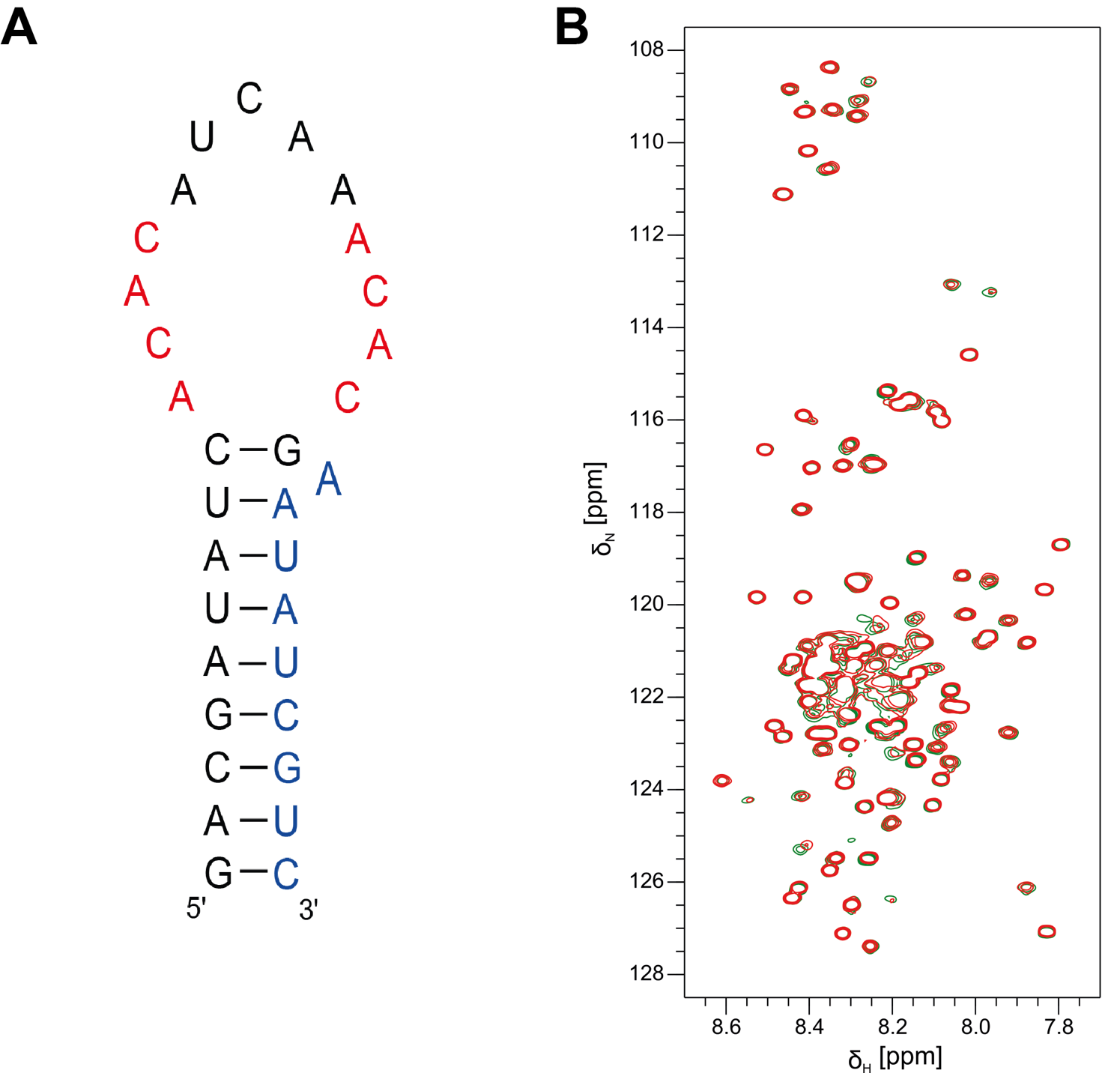
**

**Supplementary Figure 6. A.** Secondary structure of SOLE RNA MSL mutant. The nucleotides in red are mutated with respect to wild-type 32mer SOLE RNA. **B.** Overlay of ^15^N-HSQC spectra of 30 μM PYM^1–160^ in the presence of 2 molar equivalents of either 32mer SOLE RNA (red) or 32mer SOLE RNA MSL mutant (green).

**
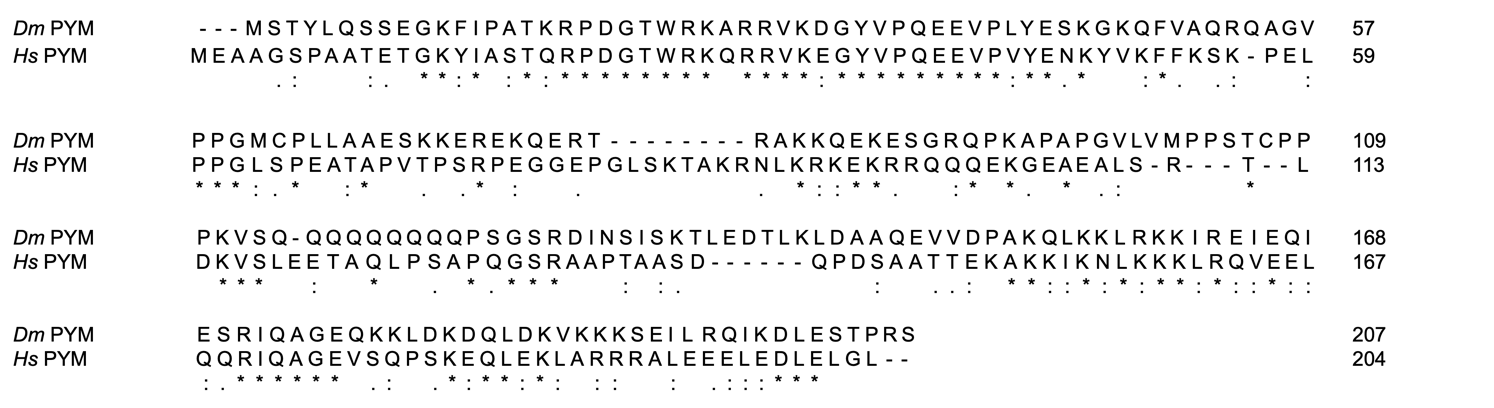
Supplementary Figure 7.** Sequence alignment of the protein PYM from *Drosophila melanogaster* (*Dm*) and *Homo sapiens* (*Hs*). “*” indicates amino acid identity; “:” indicates side chains with strongly similar properties; “.” indicates side chains with weakly similar properties.
